# Mindfulness-Based Mobile Apps and Their Impact on Well-Being in Nonclinical Populations: Systematic Review of Randomized Controlled Trials

**DOI:** 10.2196/44638

**Published:** 2023-08-04

**Authors:** Katrin Schwartz, Fabienne Marie Ganster, Ulrich S Tran

**Affiliations:** 1 Department of Cognition, Emotion, and Methods in Psychology Faculty of Psychology University of Vienna Vienna Austria

**Keywords:** mindfulness, well-being, mobile app, systematic review, randomized controlled trial, RCT, mobile phone

## Abstract

**Background:**

Mindfulness-based mobile apps have become popular tools for enhancing well-being in today’s fast-paced world. Their ability to reduce geographical, financial, and social barriers makes them a promising alternative to traditional interventions.

**Objective:**

As most available apps lack a theoretical framework, this review aimed to evaluate their effectiveness and assess their quality. We expected to find small sample sizes, high dropout rates, and small effect sizes in the included studies.

**Methods:**

A systematic literature search was conducted using PsycInfo, PsycNet, PubMed, an institutional search engine (u:search), and Google Scholar. Randomized controlled trials assessing the impact of mobile mindfulness apps on well-being in nonclinical samples were included. Study selection, risk of bias (using the version 2 of the Cochrane risk-of-bias tool for randomized trials), and reporting quality (using selected CONSORT [Consolidated Standards of Reporting Trials] statement criteria) assessments were performed by 2 authors independently and discussed until a consensus was reached.

**Results:**

The 28 included randomized controlled trials differed in well-being measures, apps, and intervention duration (7 to 56 days; median duration 28 days). A wide range of sample sizes (12 to 2282; median 161) and attrition rates (0% to 84.7%; median rate 23.4%) were observed. Most studies (19/28, 68%) reported positive effects on at least one aspect of well-being. The effects were presented using different metrics but were primarily small or small to medium in size. Overall risk of bias was mostly high.

**Conclusions:**

The wide range of sample sizes, attrition rates, and intervention periods and the variation in well-being measures and mobile apps contributed to the limited comparability of the studies. Although most studies (16/28, 57%) reported small or small to medium effects for at least one well-being outcome, this review demonstrates that the generalizability of the results is limited. Further research is needed to obtain more consistent conclusions regarding the impact of mindfulness-based mobile apps on well-being in nonclinical populations.

## Introduction

### Background

Mindfulness has its roots in Buddhism [1] and has become a popular field of research in psychology [2]. There has been an exponential rise in mindfulness meditation research over the past 2 decades [3]. Although there is not yet a clear definition [4], an operational working definition describes mindfulness as “the awareness that emerges through paying attention on purpose, in the present moment, and nonjudgmentally to the unfolding of experience moment by moment” [5]. By helping individuals disengage from automatic thoughts, habits, and unhealthy behavior patterns, mindfulness may play an important role in fostering behavioral regulation [6] and, thus, is believed to promote well-being [7].

Well-being is a complex construct consisting of several factors [8]. As with mindfulness, there is no consensus definition, so different well-being definitions include different descriptions, components, and determinants [9]. In this review, we define well-being not as the absence of illness but use the definition by Diener [10] that incorporates positive and negative affect as well as life satisfaction and flourishing [11]. Many studies have found that mindfulness-based interventions have a strong influence on well-being [12] and are believed to improve mental health in clinical as well as nonclinical populations [13,14]. The term *nonclinical* refers to individuals in good health or whose symptoms are below the threshold for mental disorders [15,16].

To date, there is a great body of research supporting the benefits of widespread mindfulness-based interventions, such as mindfulness-based stress reduction [17] and mindfulness-based cognitive therapy [18]. These interventions are associated with several beneficial effects, including reductions in negative affect and increases in positive affect and life satisfaction [13,14]. Nevertheless, some limitations to these programs also need to be considered. Not only are they 8 weeks long, consist of group-based interventions, and require up to 45 minutes of daily home practice, but they are also expensive and may put individuals in uncomfortable situations by having to expose themselves [19,20]. All these are potential barriers to their use.

A possible solution to reduce these barriers is offered by the booming field of mindfulness-based mobile apps (MBMAs) [13,21], where mindfulness content is mostly delivered via audio-guided meditation. MBMAs are more easily accessible, diverse, flexible, dynamic, discreet, and cheaper than conventional mindfulness-based interventions [22]. Owing to their portable nature, interventions delivered via mobile phone can reduce geographical, social, and financial barriers [23] and, thus, have the potential to reach a wide range of people [24]. Even though the market for mindfulness meditation apps has grown exponentially in the last few years [25], the theoretical framework aimed at delivering such interventions lacks evidence [22]. Only a small number have been scientifically proven to be efficient in providing support for an increase in well-being [25].

As mental health problems have become one of the leading issues of today’s population, often leading to long-term disability in the Western world [26,27], it is essential to offer convenient services to prevent the emergence of disorders (for a review of the effectiveness of prevention, see, eg, the study by Cuijpers et al [28]). There are numerous reviews and meta-analyses that deal with different disorders in clinical samples [29-31], but only a small share of the extant literature focuses on the potential positive impact of MBMAs on well-being in nonclinical samples. Although a recent meta-analysis [32] showed the importance of mobile-based mindfulness interventions for depressive, anxiety, and stress symptoms, this review fills the gap in research on well-being outcomes. Available studies suggest that short mindfulness meditations via smartphone apps are able to improve mental health in nonclinical populations [24]. However, studies are often characterized by small sample sizes, sometimes because of high attrition rates (eg, Economides et al [19] and Walsh et al [33]). In addition, studies use different definitions and measures of well-being, making it hard to compare the reported results. To our knowledge, no review to date has provided a comprehensive overview of the studies in this field of research.

### Objectives

Although smartphone apps may provide an easy and cheap alternative to traditional mindfulness programs, caution is advised as most available apps still lack evidence of effectiveness [25]. Therefore, the primary goal of this systematic review was to provide an overview of the extant research on the impact of MBMAs on well-being in nonclinical samples. Furthermore, we aimed to critically examine the quality of reporting and risk of bias in this field of research by applying the version 2 of the Cochrane risk-of-bias tool for randomized trials (RoB 2) [34] and a selection of CONSORT (Consolidated Standards of Reporting Trials) statement [35] criteria. We hypothesized that extant studies were characterized by small sample sizes (hypothesis 1) and high attrition rates (hypothesis 2) and expected to find only small effect sizes (hypothesis 3) [36] regarding well-being.

## Methods

### Search Strategy

The systematic literature search used the publicly available databases PsycInfo, PsycNet, and PubMed. In addition to these databases, the search engine Google Scholar and an institutional search engine at the University of Vienna, u:search, were used. The following search terms were used for all databases on February 28, 2023: “mindful*” AND “well-being” OR “wellbeing” OR “well being” AND “rct” OR “randomized controlled trial” OR “randomise* control* trial” AND “app” OR “mobile app” OR “apps” OR “mobile device applications” OR “mobile apps” OR “smartphone.” The term “mindful*” was complemented with “mindfulness” in u:search as the search string with the former term retrieved no studies there. As several relevant papers did not include the terms “mindfulness” or “well-being” in the title, it was also decided to conduct searches without these terms using the following alternative search string: “well-being” OR “wellbeing” OR “well being” AND “rct” OR “randomized controlled trial” OR “randomise* control* trial” AND “app” OR “mobile app” OR “mobile” OR “apps” OR “mobile device applications” OR “mobile apps” OR “smartphone” as well as “mindful*” AND “rct” OR “randomized controlled trial” OR “randomise* control* trial” AND “app” OR “mobile app” OR “mobile” OR “apps” OR “mobile device applications” OR “mobile apps” OR “smartphone.” We conducted 5 searches to increase the chances of finding newly published papers.

Following the advice of one of the reviewers, a further literature search was performed on May 6, 2023, using individually tailored search strategies for each database. To minimize possible selection bias, validated filters [37-39] were used in PubMed and PsycInfo for eligible study designs (randomized controlled trials [RCTs]), and the outcome measure criterion (see the following section) was only applied during screening. The final search string for PubMed was “(mindful*) AND (app OR mobile app OR apps OR mobile device applications OR mobile apps OR smartphone) AND (randomized controlled trial [Publication Type] OR randomized[Title/Abstract] OR placebo[Title/Abstract]).” The final search term for PsycInfo was “(mindful*) AND (app OR mobile app OR apps OR mobile device applications OR mobile apps OR smartphone) AND (TX double-blind OR TX random: assigned OR TX control).” No validated filter was available for PsycNet, which is why both the study design criterion and the outcome measure criterion were applied only during screening to minimize selection bias. The final search term for PsycNet was “(mindful*) AND (app OR mobile app OR apps OR mobile device applications OR mobile apps OR smartphone).”

We searched for studies in English and German, and only papers in English turned out to meet the inclusion criteria (see the following section). All relevant studies were included independent of their publication date. In total, 2 studies were retrieved by searching the reference lists of other relevant papers and were also included as they met all the inclusion criteria.

### Selection of Studies

The searches in the databases, as well as the screening of titles, abstracts, and full articles, were conducted by 2 authors independently. After removing duplicates, the titles and abstracts of all the remaining studies were screened for relevance, and studies that did not meet the inclusion criteria were discarded. The predefined inclusion criteria are presented in Textbox 1. The remaining papers were downloaded and carefully reviewed. Both authors independently assessed the eligibility of the studies by using the predefined inclusion and exclusion criteria. Uncertainties or disagreements were discussed between the 2 authors and with the third author of this study until a consensus was reached.

**Predefined inclusion and exclusion criteria.**

**Scientific field of interest**

- Inclusion criteria: mindfulness
- Exclusion criteria: no explicit investigation of mindfulness

**Article type**

- Inclusion criteria: randomized controlled trial
- Exclusion criteria: no randomization, less than at least 2 study arms, no control condition, and no specific manipulation of variables

**Outcome measure**

- Inclusion criteria: well-being (general well-being, positive affect, negative affect, life satisfaction, and flourishing)
- Exclusion criteria: no standardized assessment of predefined aspects of well-being

**How content was provided**

- Inclusion criteria: smartphone app
- Exclusion criteria: in person, websites, group interventions, educational literature, and podcasts

**Population**

- Inclusion criteria: nonclinical
- Exclusion criteria: clinical

**Language**

- Inclusion criteria: English or German
- Exclusion criteria: every other language

### Data Items, Quality Assessment, and Coding of Studies

The following information was coded from the included studies: population, sample size of the intervention and control groups, and attrition rate after randomization. Furthermore, we coded the apps used and control conditions, number of sessions, duration of the sessions in minutes, total duration of the interventions, well-being measures used, and stated effect sizes reported. In total, 2 authors independently rated the risk of bias using the Cochrane RoB 2 [34] and assessed the quality of reporting using a selection of the CONSORT statement [35] criteria. The RoB 2 allows for risk of bias ratings in the following aspects: randomization process, deviations from the intended interventions, missing outcome data, measurement of the outcome, selection of the reported result, and overall bias. Each domain contains signaling questions that were answered using the following response options: *yes*, *probably yes*, *probably no*, *no*, and *no information*. After the authors independently rated the single domains, the implemented algorithm was used to decide on the final risk of bias, the scores being *low risk*, *some concerns*, or *high risk*. In the case of different results, assessments were discussed by the 2 raters and with the third author of this study until a consensus was reached.

From the CONSORT statement criteria, we chose the most informative and relevant points of the reporting quality of the included RCTs, which were identification as a randomized trial in the title; structured summary of trial design, methods, results, and conclusions; specific objectives or hypotheses; eligibility criteria for participants; the interventions for each group, with sufficient details to allow for replication, including how and when they were actually administered; completely defined prespecified primary and secondary outcome measures, including how and when they were assessed; how the sample size was determined; method used to generate the random allocation sequence; type of randomization and details of any restriction; who generated the random allocation sequence, who enrolled participants, and who assigned participants to interventions; if done, who was blinded after assignment to interventions; for each group, the number of participants who were randomly assigned, received intended treatment, and were analyzed for the primary outcome; for each group, losses and exclusions after randomization, together with reasons; a table showing baseline demographic and clinical characteristics for each group; for each primary and secondary outcome, the results for each group, the estimated effect size, and its precision; results of any other analyses performed, including subgroup analyses and adjusted analyses, distinguishing prespecified from exploratory; trial limitations, addressing sources of potential bias, imprecision, and multiplicity of analyses if relevant; generalizability of the trial findings; interpretation consistent with results, balancing benefits and harms, and considering other relevant evidence; registration number and name of trial registry; and where the full trial protocol could be accessed, if available.

For the classification of the degree of fulfillment, 3 gradings were used. The term *stated* means that sufficient information was provided. *Partly stated* or *partly given* were used when information was only partially available. For example, Economides et al [19] mentioned their outcome measures but did not differentiate between primary and secondary, only fulfilling the associated CONSORT point to some extent. In case of missing information, we settled for the term *not stated*. In the rare cases of different results, assessments were discussed by the 2 raters and the third author of this study until a consensus was reached.

## Results

### Study Characteristics

**Figure 1:**
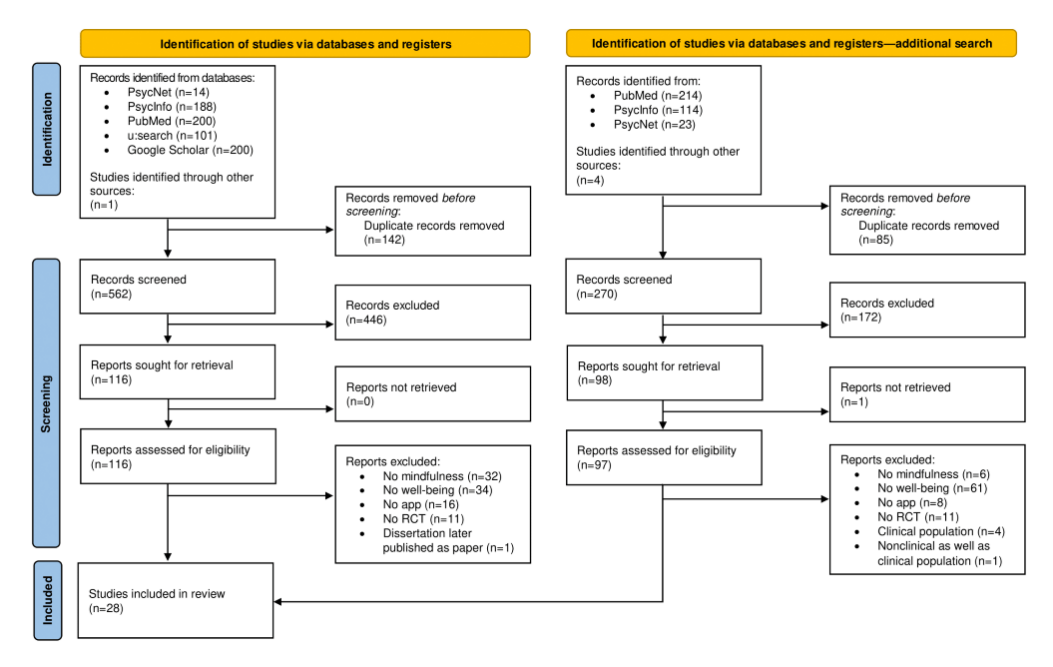
PRISMA (Preferred Reporting Items for Systematic Reviews and Meta-Analyses) flowchart of study selection. The search results totaled 2613 in PubMed and >22,000 in Google Scholar. We screened the first 200 hits in each of these databases, ranked by relevance. For the study that could not be retrieved, the researcher was contacted via email but never answered. RCT: randomized controlled trial.

A total of 28 studies were included in this review. The study selection process is presented in a PRISMA (Preferred Reporting Items for Systematic Reviews and Meta-Analyses) flowchart [40] in Figure 1. A detailed overview of the included studies is provided in Table 1.

**Table 1:** Information about the included studies (N=28)^a^.

Study	Population	App (number of participants)	Control (number of participants)	Attrition rate after randomization until latest time of assessment (%)	Number of sessions and duration of sessions in minutes	Duration of intervention	Well-being scale	Reported effect sizes (95% CIs if applicable)
Bostock et al [41], 2019	Employees	Headspace (128)	Waitlist (110)	21.85^b^	45; 10-20	8 weeks	WEMWBS^c^	Time×group interaction: Well-being: η_p_^2^=0.037; Positive affect: η_p_^2^=0.04
Carissoli et al [42], 2017	Pregnant women	BenEssereMamma (35)	Childbirth class (43)	—^d^	20; free to choose	4 weeks	PWB^e^	—
Champion et al [43], 2018	General population	Headspace (38)	Waitlist (36)	16.22^f^	30; 10-20	30 days	SWLS^g^	Cohen *d*=0.60 (0.08-1.12)
Coelhoso et al [25], 2019	Female hospital employees	Self-developed mindfulness app (250)	Self-developed app, monitoring of perceptions (240)	54^b,f^	36; 15	8 weeks	WHO-5^h^	Time×group interaction: η_p_^2^=0. 047
Deady et al [44], 2022	Employees	HeadGear (1128)	Mood monitoring app (1143)	81.36^f^	30; 5-10	30 days	WHO-5	—
Economides et al [19], 2018	General population	Headspace (mindfulness content; 87)	Headspace (audiobook; 82)	56.88^f^	10; 10	1 month	SPANE^i^	Cohen *d*=0.47 (−1.92 to 2.87)
Flett et al [24], 2018	University students	Headspace (72) and Smiling Mind (63)	Evernote (75)	17.7^b^; 8.57^f^	10; 10	10 days	Flourishing Scale	After the intervention: Headspace: Hedges *g*=0.08; Smiling Mind: Hedges *g*=0.12. Follow-up: Headspace: Hedges *g*=0.01; Smiling Mind: Hedges *g*=−0.07
Fuller-Tyszkiewicz et al [45], 2020	Caregivers	StressLess (73)	StressMonitor (110)	37.16^f^	Not reported	5 weeks	PWI^j^	Within-group: Baseline–after the intervention: StressLess: Cohen *d*=0.135; StressMonitor: Cohen *d*=0.428 (subjective well-being worsened). After the intervention–follow-up: StressLess: Cohen *d*=0.621; Emotional well-being: Cohen *d*=0.742
Gnanapragasam et al [46], 2023	Health care workers	Foundations (502)	Waitlist (500)	10.78^f^	28; duration not reported	4 weeks	SWEMWBS^k^	Cohen *d*=0.14 (0.05-0.22)
Hirshberg et al [47], 2021	School employees	Healthy minds program (346)	Waitlist (320)	13.06^f^	10 lessons+14 guided meditations; 5-30	4 weeks	WHO-5	After the intervention: Cohen *d*=0.42 (0.27-0.58). Follow-up: Cohen *d*=0.34 (0.19-0.49)
Howells et al [22], 2016	General population	Headspace (97)	Catch notes (list-making app; 97)	37.95; 37.63^f^	10; 10	10 days	SWLS, Flourishing Scale, and PANAS^l^	Time×group interaction: SWLS: η_p_^2^=0.003; Flourishing: η_p_^2^=0.006; Positive affect: η_p_^2^=0.071; Negative affect: η_p_^2^=0.010
Keng et al [48], 2022	Health care workers	Headspace (40)	Lumosity (40)	1.25^f^	21; 10	3 weeks	PWI	After the intervention: *f*^2^=0.03. Follow-up: *f*^2^=0.06
Levin et al [49], 2022	University students	Stop, Breathe, and Think (10)	Waitlist (13)	30.43^f^	28; 1-10	4 weeks	MHC-SF^m^	Hedges *g*=0.52 (90% CI −0.31 to 1.41)
Lindsay et al [50], 2018	General population	Self-developed app (MA^n^; 58) and MO^o^ (58)	Coping control (37)	2^b^; 5.88^f^	14; 20	2 weeks	State positive and negative affect (momentary and diary assessments)	Positive affect: MA versus MO: Hedges *g*=0.46; MA versus control: Hedges *g*=0.71; MO versus control: Hedges *g*=0.25. Momentary positive affect: MA versus MO: Hedges *g*=0.41; MA versus control: Hedges *g*=0.66; MO versus control: Hedges *g*=0.25. Within-group negative affect: MA: Cohen *d*=0.40; MO: Cohen *d*=0.36; Control: Cohen *d*=0.12. Within-group momentary negative affect: MA: Cohen *d*=0.38; MO: Cohen *d*=0.41; Control: Cohen *d*=0.24
Mak et al [51], 2018	General population	Living With Heart MBP^p^ (739); Living With Heart SCP^q^ (748)	Living With Heart cognitive behavioral psychoeducation program (795)	84.71^f^	28; 10-15	4 weeks	WHO-5	Within-group after the intervention: MBP: Cohen *d*=0.31; SCP: Cohen *d*=0.40; Control: Cohen *d*=0.36. Within-group follow-up: MBP: Cohen *d*=0.51; SCP: Cohen *d*=0.40; Control: Cohen *d*=0.38
Noone and Hogan [52], 2018	University students	Headspace mindfulness meditation (43)	Headspace sham meditation (48)	21.98^f^	30; 10	6 weeks	PANAS and WEMWBS	—
Ponzo et al [53], 2020	University students	BioBase (130)	Waitlist (132)	53.1^f^	42; 5	4 weeks	WEMWBS	Within-group after the intervention: BioBase: Cohen *d*=0.65; Waitlist: Cohen *d*=0.15. Follow-up: BioBase: Cohen *d*=1.16; Waitlist: Cohen *d*=0.38
Robinson [54], 2018	Community support workers	Headspace (8)	Waitlist (4)	0^f^	30; 10	30 days	PANAS	Within-group positive affect: Headspace: η^2^=0.181; Waitlist: η^2^=0.614. Negative affect: Headspace: η^2^=0.054; Waitlist: η^2^=0.54
Schulte-Frankenfeld and Trautwein [55], 2021	University students	Balloon (50)	Waitlist (49)	35.35^f^	57; 10	8 weeks	LSS^r^	η_p_^2^=0.032. Time×group interaction: η_p_^2^=0.034
Smith et al [56], 2020	Employees	Boosts (107)	Waitlist (108)	21.4^f^	28; 6-9	4 weeks	PANAS	Within-group: Boosts: β=.23 (−.36 to −.10); Waitlist: β=−.12 (−.23 to −.01)
Taylor et al [57], 2022	Health care workers	Headspace (1095)	Moodzone (1087)	48.53^f^	30; 10	30 days	SWEMWBS	After the intervention: Hedges *g*=0.07. Follow-up: Hedges *g*=0.19
Thabrew et al [58], 2022	General population	Whitu: seven ways in seven days (45)	Waitlist (45)	8.8^f^	Not reported	4 weeks	SWEMWBS and WHO-5	Within-group: WHO-5: *f*^2^=0.05; WEMWBS: *f*^2^=0.077
Vu [59], 2018 (pilot study)	University students	Pacifica (full intervention; 21)	Pacifica Lite (20)	17.07^f^	7; duration not reported	1 week	PANAS-SF^s^ and PROMIS^t^	PANAS: Negative affect: η_p_^2^=0.12; Positive affect: η_p_^2^=0.01; Global mental health: η_p_^2^=0.08. PROMIS: Negative affect: Cohen *d*=−0.94; Positive affect: Cohen *d*=−0.60; Global mental health: Cohen *d*=0.38
Vu [59], 2018	University students	Pacifica (full intervention; 140)	Pacifica Lite (138); waitlist (142)	24.76^f^	14; duration not reported	2 weeks	PANAS-SF and PROMIS	Negative affect: Pacifica versus waitlist: Cohen *d*=−0.23; Pacifica versus Lite: Cohen *d*=−0.30; Lite versus waitlist: Cohen *d*=0.07. Positive affect: Pacifica versus waitlist: Cohen *d*=−0.07; Pacifica versus Lite: Cohen *d*=−0.02. Lite versus waitlist: Cohen *d*=−0.04. Global mental health: Pacifica versus waitlist: Cohen *d*=0.2; Pacifica versus Lite: Cohen *d*=0.11; Lite versus waitlist: Cohen *d*=0.11
Walsh et al [33], 2019	University students	Wildflowers (45)	Mobile game “2048” (41)	20.37^f^ (postintervention measures); 26.85^f^ (state measures)	21; 10	3 weeks	PWBS^u^	Acceptance: *r*=0.15; Awareness: *r*=0.14; Openness: *r*=0.26; Alerting effect: *r*=−0.05; Orienting effect: *r*=−0.05; Conflict monitoring: *r*=0.15. Time×group interaction: Acceptance: *r*=0.21; Awareness: *r*=0.10; Openness: *r*=−0.05; Alerting effect: *r*=0.15; Orienting effect: *r*=0.15; Conflict monitoring: *r*=−0.24
Xu et al [60], 2022	Emergency department staff	Headspace (74)	Waitlist (74)	35.14^f^	28; 10	4 weeks	WEMWBS	Within-group after the intervention: Headspace: Cohen *d*=0.56; Waitlist: Cohen *d*=0.49. Follow-up: Headspace: Cohen *d*=0.48; Waitlist: Cohen *d*=0.51
Yang et al [61], 2018	Medical students	Headspace (45)	Waitlist (43)	Not reported	30; 10-20	30 days	GWBS^v^	—
Yoon et al [62], 2022	Stressed employees	InMind (22)	Waitlist (23)	2.22^f^	28; 20	4 weeks	COMOSWB^w^	Within-group baseline–after the intervention: Cohen *d*=0.54; Baseline–follow-up: Cohen *d*=0.51. After the intervention–follow-up: Cohen *d*=0.07; Group×time interaction: η^2^=0.090

^a^If not stated otherwise in the table, effect sizes are for between-group comparisons.

^b^Attrition rates as reported in the study.

^c^WEMWBS: Warwick-Edinburgh Mental Well-being Scale.

^d^Not available.

^e^PWB: the Italian version of the psychological well-being questionnaire.

^f^Attrition rates as calculated by the current authors (based on the reported numbers in the studies).

^g^SWLS: Satisfaction with Life Scale.

^h^WHO-5: World Health Organization 5-item Well-Being Index.

^i^SPANE: Scale of Positive and Negative Experience.

^j^PWI: Personal Wellbeing Index.

^k^SWEMWBS: Warwick-Edinburgh Mental Well-being Scale–Short Version.

^l^PANAS: Positive and Negative Affect Schedule.

^m^MHC-SF: Mental Health Continuum–Short Form.

^n^MA: monitor+accept.

^o^MO: monitor only.

^p^MBP: mindfulness-based program.

^q^SCP: self-compassion program.

^r^LSS: Questionnaire for the Assessment of Happiness (*Lebensglückskala* in German; the study was conducted with German-speaking participants).

^s^PANAS-SF: Positive and Negative Affect Schedule–Short-Form.

^t^PROMIS: Patient-Reported Outcome Measurement Information System.

^u^PWBS: Psychological Wellbeing Scale.

^v^GWBS: General Well-Being Schedule.

^w^COMOSWB: Concise Measure of Subjective Well-Being.

The included studies provided evidence for 18 different apps. In total, 39% (11/28) of the studies used Headspace [63], which was thus the single most used app. Other studies (17/28, 61%) used the following apps: HeadGear [44]; Smiling Mind [24]; Healthy Minds program [47]; Stop, Breathe, and Think [49]; Living With Heart [51]; Balloon [55]; Pacifica [59]; Wildflowers [33]; BioBase [53]; Whitu [58]; Foundations [46]; BenEssereMamma [42]; StressLess [45]; Boosts [56]; InMind [62]; and 2 self-developed apps [25,50] (ie, apps that were programmed for the study and are not commercially available in app stores). A total of 11% (3/28) of the studies investigated >1 intervention [24,50,51]. Although most apps conveyed mindfulness content solely through audio-guided meditations (eg, Economides et al [19]), including exercises such as the body scan (eg, Mak et al [51]), breathing techniques, or the practice of nonjudgment of emotions (eg, Flett et al [24]) with the common goal of grounding awareness in the present moment [55], others also implemented educational audio or video lessons aiming to explain the rationale of mindfulness (eg, Bostock et al [41] and Champion et al [43]). The intervention periods lasted from 1 week to a maximum of 8 weeks. The number of sessions to be completed by the participants ranged from 7 to 57, with a duration ranging from 1 to 30 minutes. In total, 4% (1/28) of the studies did not state instructions on the number of sessions to be completed or their duration [45].

For the assessment of well-being, various scales were used. The most common was the World Health Organization 5-item Well-Being Index [64], measuring general well-being. Other scales used for the assessment of general well-being were the Warwick-Edinburgh Mental Well-being Scale [65] as well as its short version [66], the Psychological Wellbeing Scale [67], the Patient-Reported Outcome Measurement Information System [68], the Italian version of the Psychological Wellbeing questionnaire [69], the Personal Wellbeing Index [70], the Scale of Positive and Negative Experience [11], and the Concise Measure of Subjective Well-Being [71]. In addition, some studies (9/28, 32%) measured individual aspects of well-being using the Positive and Negative Affect Schedule [72], Satisfaction with Life Scale [73], Questionnaire for the Assessment of Happiness [74], and Flourishing Scale [11].

Concerning control conditions, 64% (18/28) of the studies did not implement any active control conditions, meaning that participants in the control groups did not complete any active interventions. Thus, these waitlist control conditions did not control for the digital placebo effect, which may lead to improvements in mental health only because of downloading and using an app [75]. Other studies used mobile apps that did not offer mindfulness meditations (eg, Flett et al [24]), educational programs (eg, Mak et al [51]), or mobile games [33] as active control conditions.

### Impact on Well-Being

The primary aim of this review was to investigate whether the use of MBMAs has an impact on well-being in nonclinical populations. In this regard, ambiguous results were found.

Substantial improvements in at least one aspect of well-being were reported in 68% (19/28) of the RCTs [19,22,25,33,41,43,45-48,50,51,53,56-60,62], indicating that participating in the mobile mindfulness intervention enhanced well-being. Although 7% (2/28) of the studies found significant results for positive and negative affect [19,50], others demonstrated changes solely in individual aspects. For example, Howells et al [22] reported increases in positive affect, whereas Vu [59] reported decreases in negative affect. Using the Warwick-Edinburgh Mental Well-being Scale and its short version, 21% (6/28) of the studies reported significant improvements in mental well-being [41,46,53,57,58,60]. For satisfaction with life, contradictory results were reported. Only 4% (1/28) of the studies reported a significant time×group interaction [43]; effects with other measures of satisfaction with life in other studies did not reach significance [22,55]. There were no significant changes in flourishing [22,24]. Finally, 18% (5/28) of the RCTs reported no significant changes in well-being outcomes at all [24,49,52,55,61].

### Sample Size, Attrition Rates, and Size of Reported Effects

We expected small sample sizes for our first hypothesis (hypothesis 1). The total sample size before attrition ranged from 12 [54] to 2282 [51], with a median of 161. Participants were mostly evenly allocated to the intervention and control groups (Table 1). A total of 68% (19/28) of the studies stated how the sample size was determined, although not a single RCT used effect sizes of well-being measures in nonclinical populations in their calculations. Only 4% (1/28) of the studies [58] used the effect sizes of well-being measures but taken out of a clinical context and concerning web-based interventions. Another study [48] based its sample size calculations on meta-analytic results concerning web-based mindfulness-based interventions [76]. However, it remained unclear whether this involved well-being as an outcome of interest. In addition, the meta-analysis examined web-based interventions rather than mobile apps.

High attrition rates were expected for our second hypothesis (hypothesis 2). We calculated dropout rates from 0% [54] to 84.7% [51], with a median of 23.4%. In total, 11% (3/28) of the studies showed discrepancies in the reported attrition rate and the rate calculated by the authors of this review using the reported numbers in these studies [22,24,50]. A total of 79% (22/28) of the studies did not state their attrition rates. It is worth noting that the highest attrition rate was observed for the largest sample [51]. We calculated a median of 106 for the total sample size after attrition, with a minimum of 12 and a maximum of 894 participants. In line with the RoB 2 [34], a difference of ≥5% in the attrition rates of the study arms was considered substantial. This was the case in 50% (14/28) of the studies. In total, 39% (11/28) of the studies [22,43,46,47,49,50,52,55,56,58,59] had significantly higher attrition in the intervention arm, whereas 11% (3/28) of the studies [19,41,45] had higher attrition in the control condition. In total, 27% (3/11) of the studies in the former group and 67% (2/3) of the latter studies concerned the app Headspace. A table containing an overview of these differences in attrition rate is provided in Table S1 in Multimedia Appendix 1 [19,22,24,25,33,41-62].

We expected to find small effect sizes regarding well-being outcomes for our third hypothesis (hypothesis 3). To account for the difference in reported effect size metrics, we decided to summarize the effects according to widely used benchmarks rather than their actual values (these are provided in Table 1) to allow for better comparability. According to Cohen [36], a Cohen *d* or Hedges *g* of 0.2 was classified as a small effect, 0.5 was classified as a medium effect, and 0.8 was classified as a large effect; a Cohen *f*^2^ of 0.02 was classified as a small effect, 0.15 was classified as a medium effect, and 0.35 was classified as a large effect; a partial η_p_^2^ [77] of 0.01 was classified as a small effect, 0.06 was classified as a medium effect, and 0.14 was classified as a large effect; and a Pearson *r* of 0.1 was classified as a small effect, 0.3 was classified as a medium effect, and 0.5 was classified as a large effect. According to the benchmarks for the standardized regression coefficient β, effects between .10 and .29 are classified as small, between .30 and .49 are classified as medium, and effects of ≥.5 are considered large. A total of 39% (11/28) of the included studies reported small effects [24,41,45,46,48,55-59,62], 29% (8/28) reported small to medium effects [19,25,33,45,47,54,58,62], and 14% (4/28) reported medium-sized effects [43,49,53,62]. In total, 29% (8/28) of the papers reported small- as well as medium-sized effects [22,45,50,51,53,59,60,62] depending on the measured aspect of well-being. Only 11% (3/28) of the studies reported large effects, namely on negative affect [59], positive affect [54], and general well-being [53]. It is worth mentioning that 4% (1/28) of the studies reporting the largest effects [54] also had the smallest sample size of all the included studies (n=12). A total of 14% (4/28) of the RCTs did not report any effect sizes at all [42,44,52,61].

### Risk of Bias and Quality of Reporting

**Figure 2:**
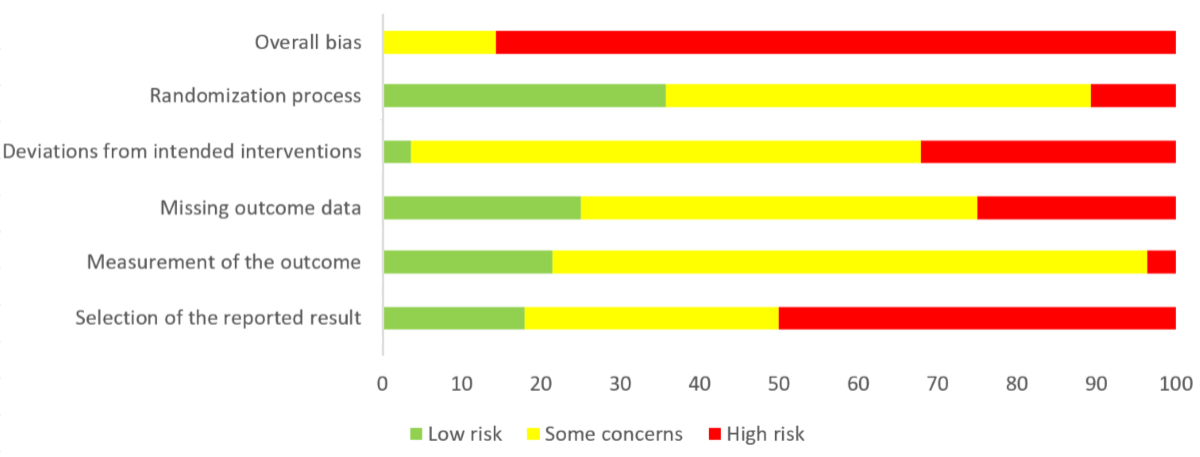
Cochrane risk of bias summary of the included randomized controlled trials.

Overall risk of bias was high for 86% (24/28) of the studies, and there were *some concerns* for the remaining 14% (4/28) of the studies (see Figure 2 for a summary of overall and individual domain ratings; a more detailed overview is provided in Figure S1 in Multimedia Appendix 1). None of the studies received a low overall risk of bias rating. Of the individual domains, the reporting of studies on the randomization process appeared to be least problematic in comparison with the other domains and most problematic concerning the domain of selection of the reported result.

**Figure 3:**
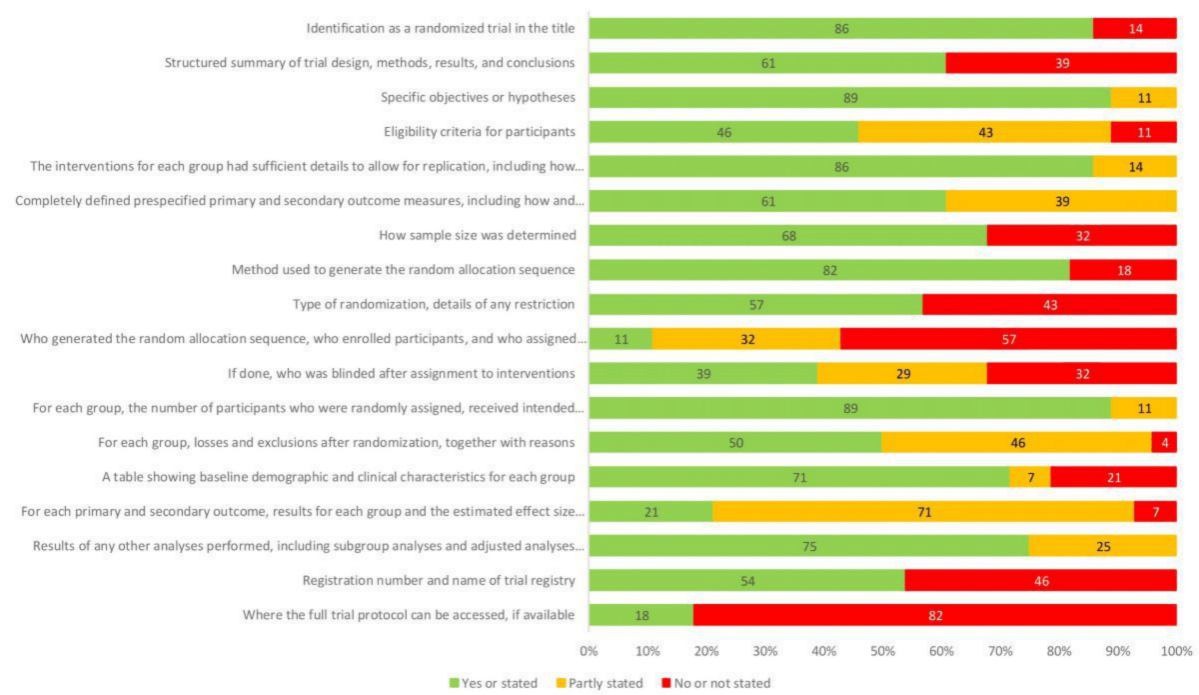
Quality of reporting using selected CONSORT (Consolidated Standards of Reporting Trials) statement criteria. Regarding the criterion “Results of any other analyses performed,” studies that did not perform any other analyses were excluded from this calculation.

**Figure 4:**
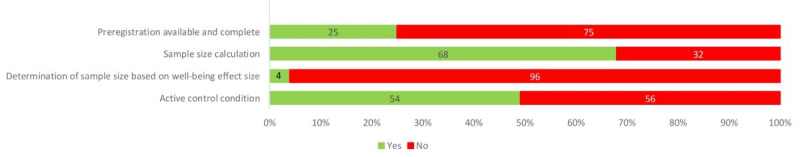
Most salient trial limitations and their degree of fulfillment. Only 1 randomized controlled trial conducted sample size calculations based on well-being effect sizes from previous research, but this effect size was taken out of a clinical context concerning web-based interventions.

Detailed results on the ratings of the selected CONSORT statement criteria are presented in Table S2 in Multimedia Appendix 1. A summary is provided in Figure 3, which presents the percentages of studies that met, partly met, or did not meet each criterion. The 2 criteria that were met with the highest frequency concerned the reporting of specific objectives or hypotheses and the number of participants who were randomly assigned, received the intended treatment, and were analyzed for the primary outcome. The 2 criteria that were partly met with the highest frequency concerned the reporting of detailed results for each group in all primary and secondary outcomes and losses and exclusions after randomization for each group. The 2 criteria that were not met with the highest frequency concerned the accessibility of full trial protocols and the reporting on who generated the random allocation sequence, who enrolled the participants, and who assigned the participants to the interventions. For 2 CONSORT statement criteria, namely generalizability and interpretation, a summary through simple percentages was not possible. Instead, Figure 4 provides an overview of the most salient trial limitations and their degree of fulfillment (all individual trial limitations are provided in Table S2 in Multimedia Appendix 1).

Trial limitations, addressing sources of potential bias; imprecision; and, if relevant, multiplicity of analyses, are shown in Figure 4. Examples of limited generalizability (external validity and applicability) of the trial findings include the selection of very specific samples (eg, university students [52,55,59]). In addition, the small sample sizes contributed to limited generalizability, especially when combined with high attrition rates [19,49,55]. In total, 7% (2/28) of the studies [43,61] interpreted (partially) not significant results (*P*>.05) as significant. Therefore, the positive effects of practicing mindfulness meditation on well-being were not fully supported by their own data. In addition, another 25% (7/28) of the studies [22,33,49,54,55,57,59] made broad and general statements about the benefits of mindfulness meditation for well-being without clear reference to their exact results. However, the data indicated either only statistical trends (*P*>.05) or benefits referring only to specific aspects of well-being (eg, positive affect) instead of its multiple aspects (ie, positive and negative affect, life satisfaction, and flourishing) that were often measured in tandem. This interpretation problem was exacerbated by the use of various definitions of the concept of well-being in the included studies. Most definitions overlapped to a large degree with the definition of this review, with the scales used measuring at least one of the aforementioned 4 aspects of well-being. However, 4% (1/28) of the studies [55] used both life satisfaction and perceived stress as indicators of well-being and interpreted the significant results observed for perceived stress (but not life satisfaction) as evidence of benefits for well-being. Nevertheless, it is also worth mentioning that most studies (22/28, 79%) did embed their findings adequately into the previous literature.

## Discussion

### Principal Findings

The aim of this systematic review was to examine the impact of mindfulness-based mobile interventions on well-being in nonclinical populations. We obtained evidence from 28 RCTs on interventions that mostly consisted of audio-guided meditations aimed at fostering present-moment awareness. Few studies (6/28, 21%) also delivered audio or video lessons to explain the rationale behind mindfulness. Most of the 28 RCTs (19/28, 68%) reported significant improvements in well-being even though the effect sizes were mostly small to medium and the overall risk of bias was mostly high. In addition, a wide range of sample sizes (12 to 2282) and attrition rates (0% to 84.7%) were observed. Taking these results into consideration, findings need to be interpreted with caution.

The highest attrition rate was observed in the largest sample [51], drastically reducing the number of effective participants in this study (349 vs 2282). The median total sample size after attrition was 106 (12 to 894). A medium-sized effect (ie, Cohen *d* or Hedges *g* of 0.5) requires a total sample size of at least approximately 100 participants to achieve 80% analytical power; a small effect (ie, Cohen *d* or Hedges *g*=0.2) requires approximately 620 participants. Thus, our first hypothesis was partly confirmed as only approximately half (14/28, 50%) of the studies appeared to be adequately powered to detect the medium-sized or even smaller effects that were of relevance (see the following paragraphs for a more detailed discussion of the magnitude of the reported effects). In addition, it is worth noting that sample size calculations, if conducted, were not based on effect sizes regarding well-being measures in nonclinical populations or concerning mobile apps but, rather, were mostly related to depression (eg, Deady et al [44]), anxiety [59], and stress (eg, Economides et al [19]). Meta-analyses of web-based mindfulness-based interventions have reported inconsistent effects on these outcomes, varying from small to large [76]. Overall, this might have led researchers to expect larger effects also on well-being. This, in turn, might have increased the risk of studies being too underpowered to reliably detect the mostly smaller effects on the well-being outcomes that were of interest in this review.

In addition, attrition rates varied widely, with the lowest being 0% and the highest being 84.7%. We calculated a median of 23.4%. Prior work has reported attrition rates from 50% to 60% for research on electronic health care delivered via the internet [78] and an average attrition rate of 43.4% in mobile health interventions delivered via smartphones [79] (for face-to-face mindfulness interventions, attrition rates are approximately 19.1% on average [80]). This higher maximum and lower average rates partly confirm our second hypothesis. However, the study by Mak et al [51] illustrated that large sample sizes may go hand in hand with high attrition rates nonetheless, which ultimately might lead to low study power. This further highlights the importance of treating the results of extant studies carefully. Attrition rates further differed in half (14/28, 50%) of the studies, with 39% (11/28) of the studies reporting significantly higher attrition rates in the intervention arm than in the control condition and 11% (3/28) of the studies reporting higher attrition rates in the control condition than in the intervention arm. The reasons for this differential attrition need to be investigated in future research in more detail as this may indicate that either the usability of the investigated apps may need improvement or that users need to be made aware of unintended effects that may prompt the discontinuation of using the app or engaging in mindfulness practice. Context factors also need to be considered in this case as differential attrition for the most investigated app, Headspace, concerned both the intervention and control groups in different studies. Attrition is a problem for intervention research overall, but differential attrition seems to be less of a problem for common, non–mobile-based mindfulness interventions [80].

Of the 28 included RCTs in this review, most (16/28, 57%) found small or small to medium effect sizes for well-being outcomes, which mostly confirms our third hypothesis, in which we expected small effect sizes. In this context, it is worth mentioning that different aspects of the well-being construct were measured in these studies and that effects were reported also using different effect size metrics. Although some studies (21/28, 75%) assessed general well-being, others measured only individual aspects (positive or negative affect, life satisfaction, and flourishing). The difference in the underlying definitions of well-being as well as the variety of scales used to measure the construct might have contributed to broad and sometimes inexact interpretations. It is further worth mentioning that nonclinical populations in many studies likely already had high well-being baselines, making large improvements hard to achieve and overall less likely. This may have contributed to the relatively small effect sizes reported in the included studies. However, in addition to the heterogeneity in the measured outcomes, there were other factors that may limit the comparability of the studies in this review to a larger degree. Most studies (11/28, 39%) investigated the effects of Headspace; however, in total, 18 different apps were used. Differences in the interventions concerning the implemented number of sessions and their duration may further contribute to the limited comparability of the investigated apps and generalizability of the results. Finally, the observed differential attrition rates in the included studies suggest that many of the reported effects may be biased.

### Limitations and Future Research

This is the first systematic review of the impact of MBMAs on well-being in nonclinical populations using RCT data and focusing on the quality of reporting and risk of bias. However, some limitations need to be mentioned. First, we did not address study outcomes other than well-being, and the quality assessment was applied to measures of well-being only. Future studies may want to also address other outcomes and investigate the effects of MBMAs on these outcomes as well. In addition, we would like to recommend that authors and researchers use a consistent terminology for well-being in their studies. This would enhance clarity and contribute to readers’ understanding but would also increase the generalizability of the results. Second, trait mindfulness could serve as an effect moderator of mindfulness interventions [81], meaning that people with high scores in mindfulness might benefit less than those with low scores. Thus, future research could also perform meta-analytic calculations using the data from the primary studies and investigate baseline trait mindfulness as a possible effect moderator. As the focus of this review lies on risk of bias and reporting quality, we did not aim to provide meta-analytic calculations ourselves. However, future meta-analyses would also be well advised to consider the possible effects of dose-response relationships with the number of sessions and their duration. This review provided evidence that the dose varies widely in extant studies.

This review provided evidence of relatively high attrition rates. Future research should investigate the possible reasons for attrition to implement appropriate actions to maintain higher study participation. Future RCTs as well as reviews and meta-analyses should also apply the Mobile App Rating Scale [82] consistently over all apps used to enhance quality and comparability. Moreover, research needs to address the issue of which intervention elements might be the most effective or may boost the effects of other intervention elements. Most of the currently available studies include guided meditations but differ with regard to other intervention elements (eg, whether to provide a theoretical explanation and rationale for the effects of mindfulness). As the number of sessions and their duration vary widely, future studies should systematically test which intervention duration provides the most effective support for the promotion of well-being. Finally, using the criteria of the CONSORT statement allowed for a very detailed and extensive assessment of the quality of reporting of the included studies in this review. Therefore, this approach is recommended also for future research.

Smartphones and mobile apps are gaining popularity, and therefore, their use has far-reaching consequences. This systematic review is consistent with previous studies showing positive but small effects of MBMAs on well-being. It provides another important step in the booming field of mindfulness research, striving to optimize the usability and quality of mobile mindfulness apps. This is especially important considering that most people today own a smartphone, making them more likely to increasingly seek help through mobile apps. These have been proven to be effective in preventing mental health issues; hence, this field of research is of high importance for a great number of people in today’s fast-paced world.

### Conclusions

This systematic review showed that some mobile mindfulness interventions have a positive impact on well-being in nonclinical populations in RCT data. Nevertheless, there was a large variation in sample size and attrition rates, the effects were predominantly of only small to medium size, and the overall risk of bias was mostly high. Assessment of the quality of reporting and risk of bias revealed a lack of a priori power calculations and active control conditions. The use of different well-being measures further limited extant studies’ comparability and the generalizability of their results. Thus, these findings emphasize that there is still a need for high-quality research on mobile apps, which become more and more important in today’s modern world where smartphones are an essential component of everyday life. Even though mobile apps are easily accessible to a large segment of the population as well as cheap and discreet, more evidence is needed to reliably evaluate their potential for enhancing users’ well-being.

## Appendix

Multimedia Appendix 1 Detailed information regarding the total sample size after attrition and attrition rates within study arms, the CONSORT (Consolidated Standards of Reporting Trials) statement reporting on study quality as well as an overview of risk of bias assessment.

## Abbreviations

CONSORT: Consolidated Standards of Reporting Trials
MBMA: mindfulness-based mobile app
PRISMA: Preferred Reporting Items for Systematic Reviews and Meta-Analyses
RCT: randomized controlled trial
RoB 2: version 2 of the Cochrane risk-of-bias tool for randomized trials

## Data Availability

All data generated or analyzed during this study are included in this published article (and its supplementary information files).
