# Supplementary material for: Mindfulness-Based Mobile Apps and Their Impact on Well-Being in Nonclinical Populations: Systematic Review of Randomized Controlled Trials
Source: J Med Internet Res. 2023 Aug 4;25:e44638. doi: 10.2196/44638 (PMC10439468; doi:10.2196/44638)
Supplement: Multimedia Appendix 1 [file jmir_v25i1e44638_app1.pdf]

Multimedia Appendix

This is a Multimedia Appendix to a full manuscript published in the Journal of Medical Internet Research.
For full copyright and citation information see http://dx.doi.org/10.2196/jmir.44638

Table S1

Total sample size after attrition and attrition rates within study arms.

Author (year)	Total sample size after attrition	Drop-out rate intervention arm(s)	Drop-out rate control group
Bostock et al [41], (2019)	186	17.97%	**26.36%**
Carissoli et al [42], (2017)	No numbers reported, calculation impossible		
Champion et al [43], (2018)	62	**23.68%**	8.33%
Coelhoso et al [25], (2019)	226	53.60%	54.17%
Deady et al [44], (2022)	424	83.73%	79.02%
Economides et al [19], (2018)*	71	50.57%	**65.85%**
Flett et al [24], (2018)	192	6.94%<br>7.94%	10.67%
Fuller-Tyszkiewicz et al [45], (2020)	115	25.00%	**30.91%**
Gnanapragasam et al [46], (2023)	894	**15.34%**	6.20%
Hirshberg et al [47], (2021)	492	**34.68%**	16.88%
Howells et al [22], (2016)	121	**41.24%**	34.02%
Keng et al [48], (2022)	79	2.50%	0.00%
Levin et al [49], (2022)	16	**40.00%**	23.08%
Lindsay et al [50], (2018)	143	**5.17%**<br>**12.07%**	0.00%
Mak et al [51], (2018)	349	83.09%<br>83.96%	86.92%
Noone and Hogan [52], (2018)	71	**27.08%**	16.28%
Ponzo et al [53], (2020)	123	54.62%	52.31%
Robinson [54], (2018)	12	0.00%	0.00%
Schulte-Frankenfeld and Trautwein [55], (2021)	64	**40.00%**	30.61%
Smith et al [56], (2020)	169	**37.38%**	5.56%
Taylor et al [57], (2022)	1123	47.85%	49.22%
Thabrew et al [58], (2022)	82	**15.56%**	0.02%
Vu [59], (2018) (pilot study)	34	15.00%	19.05%
Vu [59], (2018)	316	**30.00%**<br>**26.81%**	17.61%
Walsh et al [33], (2019)	86	22.41%	18.00%
Xu et al [60], (2022)	96	36.49%	33.78%
Yang et al [61], (2018)	No numbers reported, calculation impossible		
Yoon et al [62], (2022)	44	0.00%	2.22%

Note. See main text for reference list. For studies with a difference of 5% or more in attrition between study arms, the study arm with the higher attrition rate is printed boldface. If two numbers are reported for drop-out rates, they refer to the two intervention arms in that study.
* ambiguous flow-chart makes it impossible to state exact values.

Table S2

CONSORT statement reporting on study quality.

Selected CONSORT Statement criteria	Bostock et al [41], (2019)	Carissoli et al [42], (2017)	Champion et al [43], (2018)	Coelhoso et al [25], (2019)	Deady et al [44], (2022)
Identification as a randomized trial in the title	No	No	Yes	Yes	Yes
Structured summary of trial design, methods, results, and conclusions	Yes	No	Yes	Yes	Yes
Specific objectives or hypotheses	Stated	Stated	Stated	Stated	Partly stated aim of study explained, no specific hypotheses or predictions mentioned
Eligibility criteria for participants	Exclusion criteria only	Inclusion criteria only	Exclusion criteria only	Inclusion criteria only	Inclusion and exclusion criteria stated
The interventions for each group with sufficient details to allow replication, including how and when they were actually administered	Yes	Yes	Yes	Yes, but self-developed app might make replication difficult	Yes
Completely defined pre-specified primary and secondary outcome measures, including how and when they were assessed	Partly stated primary explicitly mentioned, secondary outcomes not specified as such	Partly stated aim of study explained, no specific hypotheses or predictions mentioned; no differentiation between primary and secondary outcomes	Partly stated no differentiation between primary and secondary outcomes	Stated	Stated
How sample size was determined	–	–	Stated pilot study, so no a priori planned sample size, strived for 70 participants as recommended for pilot RCTs	Stated	Stated
Method used to generate the random allocation sequence	Stated random number generator software	–	Stated computer-generator	Stated Microsoft Excel RANDBETWEEN	Stated randomization algorithm integrated in trial management software

Type of randomization, details of any restriction	–	–	Stated<br>simple randomization	Stated<br>1:1 allocation ratio	Stated<br>block design with size of 10
Who generated the random allocation sequence, who enrolled participants, and who assigned participants to interventions	–	–	Partly stated<br>sequence generation & randomization: research team	Partly stated<br>randomization & assignment: research assistant	–
If done, who was blinded after assignment to interventions	Partly stated<br>participants were not blinded, not stated whether the research team was	–	Partly stated<br>research team was not blinded, not stated whether participants were blinded	Stated<br>participants were not blinded, outcome assessors and data analysts were blinded	Partly stated<br>participants were blinded to allocation, not mentioned whether researchers were blinded
For each group, the numbers of participants who were randomly assigned, received intended treatment, and were analyzed for the primary outcome	Stated	–	Stated	Stated	Stated
For each group, losses and exclusions after randomization, together with reasons	Partly stated<br>no reasons for drop-out at t3 given	–	Partly stated<br>reasons unknown	Stated	Stated
A table showing baseline demographic and clinical characteristics for each group	Yes	No	Yes	Yes	Yes
For each primary and secondary outcome, results for each group, and the estimated effect size and its precision (such as 95% confidence interval)	Partly stated<br>no CIs for effect sizes	–	Stated	Partly stated<br>no CIs for effect sizes	Partly stated<br>only mean differences, not results for each group, effect sizes not for all outcomes
Results of any other analyses performed, including subgroup analyses and adjusted analyses, distinguishing pre-specified from exploratory	No other analyses performed	No other analyses performed	Partly stated	No other analyses performed	No other analyses performed

Trial limitations, addressing sources of potential bias, imprecision, and, if relevant, multiplicity of analyses	- no active control condition<br>- long term effect remained unknown due to short-term follow-up<br>- did not control for personality traits that might have moderated the outcomes<br>- sample size was not calculated → might be underpowered<br>- no preregistration	- no active control condition<br>- no sample size calculations<br>- no preregistration<br>- attrition rate neither stated nor calculable due to non-transparency of drop-outs<br>- long term effect undetectable due to lack of follow-up assessments<br>- no statements about randomization process or participant allocation<br>- effect sizes not reported	- no active control condition<br>- no sample size calculated (pilot study)<br>- small sample size → probably underpowered<br>- no follow-up measures after 30 days<br>- the stated confidence that not blinding the researchers did not have an influence on the analyses cannot be guaranteed<br>- results of ANOVA for main effect intervention not mentioned → post hoc t-tests were performed → no statistical analyses were preregistered → multiplicity of analysis<br>- simple randomization has led to gender imbalance in the two study arms	- while sample size calculations were mentioned, it remains unclear which effect size was used<br>- not blinding participants increased risk of bias<br>- study does not acknowledge its own limitations which might have biased their interpretation<br>- preregistration lacks methods of statistical analyses → unclear if secondary analyses were planned in advance → multiplicity possible	- sample size calculations were based on the effect size for depression → applicability for well-being unclear<br>- mean values were not stated<br>- even though well-being was a secondary outcome that reached significance in mean differences from baseline to post intervention but not from baseline to follow-up, results were neither discussed nor interpreted<br>- possible influence through uncontrolled personality traits<br>- no effect sizes reported
Generalizability (external validity, applicability) of the trial findings	- results probability limited to healthy working population	- limited generalizability as sample consisted solely of pregnant women	- as framing of study content may have biased the sample, results need to be generalized with caution<br>- small sample sizes limits generalizability	- generalizability is limited due to only including female participants<br>- results limited to cohorts with well-being scores that are below average (even though there was a significant improvement, well-being scores were still below average after study completion)<br>- generalizability restricted as used app was self-developed and inaccessible to other cohorts	- limited generalizability due to high attrition rate (sample size was still bigger than in other studies)
Interpretation consistent with results, balancing benefits and harms, and	- inconsistent assessment of results in relation to prior studies	- interpretation consistent with results<br>- no effect sizes reported	- interpretation not consistent with results (increased life satisfaction	- while correctly reporting the significant time-effect of general well-being, it	- while finding were embedded into prior literature, results regarding

considering other relevant evidence	- did not refer to results of systematic reviews or meta-analyses when classifying outcomes<br>- results consistent with other recent studies	- results not embedded into prior literature → hinders comparability	was reported, even though t-test did not reach statistical significance)<br>- references to other studies made	wasn't mentioned that the scores were continuously higher in the control group as can be seen in Figures 3 and 4<br>- results impeded into prior literature	well-being remained unnoticed in the discussion
Registration number and name of trial registry	–	–	Stated<br>ISRCTN registry (34618894) retrospectively	Stated<br>clinicaltrials.gov (02637414)	Stated<br>Australian and New Zealand Clinical Trials Registry (ACTRN12617000548336)
Where the full trial protocol can be accessed, if available	–	–	Stated<br>link in Appendix of study	–	Stated<br>reference given

Selected CONSORT Statement criteria	Economides et al [19], (2018)	Flett et al [24], (2018)	Fuller-Tyszkiewicz et al [45], (2020)	Gnanapragasam et al [46], (2023)	Hirshberg et al [47], (2021)
Identification as a randomized trial in the title	Yes	Yes	Yes	Yes	Yes
Structured summary of trial design, methods, results, and conclusions	No	No	Yes	Yes	No
Specific objectives or hypotheses	Stated	Stated	Stated	Partly stated<br>aim of study explained, no specific hypotheses or predictions mentioned	Stated
Eligibility criteria for participants	Inclusion and exclusion criteria stated	–	Inclusion criteria only	Inclusion and exclusion criteria stated	Inclusion and exclusion criteria stated
The interventions for each group with sufficient details to allow replication, including how and when they were actually administered	Yes	Yes	Yes	Yes	Yes
Completely defined pre-specified primary and secondary outcome measures, including how and when they were assessed	Partly stated<br>no differentiation between primary and secondary outcomes	Stated	Stated	Stated	Stated
How sample size was determined	Stated	Stated	Stated	Stated	Stated
Method used to generate the random allocation sequence	Stated<br>computer generator	Stated<br>random number generator	Stated<br>Qualtrics	Stated<br>web-based system	Stated<br>random number generator
Type of randomization, details of any restriction	Stated<br>simple randomization	–	Stated<br>3:2 assignment in blocks of 5	Stated<br>block design (1:1)	Stated<br>simple random assignment (1:1)
Who generated the random allocation sequence, who enrolled participants, and who assigned participants to interventions	–	Stated<br>researcher	–	Partly stated<br>allocation sequence: research team	Partly stated<br>randomization: computer system
If done, who was blinded after assignment to interventions	–	Partly stated<br>cover story to blind participants, not stated	Stated<br>no one was blinded	Stated<br>participants were not blinded; senior statistician, trial statistician, co-	Stated<br>participants were not blinded, researchers

		whether researchers were blinded		investigators, and one chief investigator were blinded; some researches were not blinded	became aware of assignment after the fact
For each group, the numbers of participants who were randomly assigned, received intended treatment, and were analyzed for the primary outcome	Stated	Partly stated<br>flowchart contains different numbers than text	Stated	Stated	Stated
For each group, losses and exclusions after randomization, together with reasons	Partly stated<br>reasons for drop-outs unknown	Partly stated<br>reasons for drop-outs unknown	Partly stated<br>no reasons for drop-outs	Partly stated<br>reasons for drop-out unknown	Stated
A table showing baseline demographic and clinical characteristics for each group	Yes	No	Yes	Yes	Yes
For each primary and secondary outcome, results for each group, and the estimated effect size and its precision (such as 95% confidence interval)	Stated	Partly stated<br>no CIs for effect sizes	Partly stated<br>no CIs for effect sizes	Stated	Stated
Results of any other analyses performed, including subgroup analyses and adjusted analyses, distinguishing pre-specified from exploratory	Partly stated	Partly stated	Stated	Stated	Stated
Trial limitations, addressing sources of potential bias, imprecision, and, if relevant, multiplicity of analyses	- sample size calculations were based on the effect size for stress → applicability for well-being unclear<br>- due to short intervention period and no follow-up assessments possible effects might not have been observed<br>- due to missing	- sample size calculations were based on the effect size for depression, anxiety and stress → applicability for well-being unclear<br>- calculated sample size was not met → study design underpowered<br>- attrition rate calculation is not transparent → different	- while sample size calculations were mentioned, it remains unclear to which measure the effect size refers to<br>- preregistration lacks methods of statistical analyses → unclear if exploratory analyses were<br>- incomplete and inconsistent reporting of	- no active control condition<br>- unclear to which effect the power analysis refers to → applicability for well-being unclear	- no active control condition<br>- sample size calculations were based on the effect size for psychological distress → applicability for well-being unclear<br>- preregistration lacks methods of statistical analyses → unclear if

	preregistration it remains unclear whether post hoc t-tests were planned in advance	numbers of participants in flow chart and text<br>- due to short intervention period and no follow-up assessments possible effects might not have been observed<br>- preregistration lacks methods of statistical analyses → unclear if secondary analyses were planned in advance → multiplicity possible	secondary outcome measures and effect sizes<br>- preregistration lacks methods of statistical analyses → unclear if exploratory analyses were planned in advance → multiplicity possible<br>- offered incentives may have affected motivation of participants → low intrinsic motivation may have had impact on execution		secondary analyses were planned in advance → multiplicity possible<br>- offered incentives may have affected motivation of participants → low intrinsic motivation may have had impact on execution
Generalizability (external validity, applicability) of the trial findings	- even though it is stated that participants showed similar characteristics as the general population, we think generalizability is limited due to quite homogenous cohort (mostly White/Caucasian, university degree, positive expectation about meditation)<br>- the small sample size makes applicability for broader population difficult	- convenience sample of undergraduate students limits external validity	- sample mainly consistent of females which might limit external validity	- generalizability limited due to predominantly female sample	- gender and race homogeneity limits generalizability
Interpretation consistent with results, balancing benefits and harms, and considering other relevant evidence	- interpretation is consistent with results<br>- results impeded into prior literature	- interpretation consistent with results<br>- results impeded into prior literature<br>- neither Headspace nor Smiling Mind managed to increase flourishing measures significantly → mentioned in discussion and possible reasons stated	- interpretation is consistent with results<br>- results impeded into prior literature	- interpretation consistent with results but well-being outcome was barely mentioned<br>- results impeded into prior literature	- interpretation consistent with results<br>- no clear reference to existing reviews in the discussion
Registration number and name of trial registry	–	Stated<br>Australian and New Zealand Clinical Trials Registry (ACTRN 368325)	Stated<br>Australian New Zealand Clinical Trials Registry (ACTRN12616000996460)	Stated<br>EudraCT: 2021-001279-18	Stated<br>clinicaltrials.gov (04426318)

Where the full trial protocol can be accessed, if available	–	–	–	Stated<br>Lamb D, Greenberg N,<br><br>Hotopf M, Raine R, Razavi R, Bhundia R, et al. NHS CHECK: protocol for a cohort study investigating the psychosocial impact of the COVID-19 pandemic on healthcare workers. BMJ Open 2021; 11: e051687.	–

Selected CONSORT Statement criteria	Howells et al [22], (2016)	Keng et al [48], (2022)	Levin et al [49], (2022)	Lindsay et al [50], (2018)	Mak et al [51], (2018)
Identification as a randomized trial in the title	Yes	Yes	No	Yes	Yes
Structured summary of trial design, methods, results, and conclusions	No	Yes	Yes	No	Yes
Specific objectives or hypotheses	Stated	Stated	Stated	Stated	Stated
Eligibility criteria for participants	Inclusion criteria only	Inclusion and exclusion criteria stated	Inclusion criteria only	Inclusion and exclusion criteria stated	Inclusion criteria only
The interventions for each group with sufficient details to allow replication, including how and when they were actually administered	Yes	Yes	Yes	Yes, but self-developed app might make replication difficult	Yes
Completely defined pre-specified primary and secondary outcome measures, including how and when they were assessed	Partly stated<br>no differentiation between primary and secondary outcomes	Partly stated<br>no differentiation between primary and secondary outcomes	Stated	Partly stated<br>no differentiation between primary and secondary outcomes	Stated
How sample size was determined	–	Stated	–	Stated	–
Method used to generate the random allocation sequence	Stated<br>website software	Stated<br>computerized random number generator	Stated<br>Qualtrics	Stated<br>computerized random number generator	Stated<br>computer system
Type of randomization, details of any restriction	–	Stated<br>block randomization	Stated<br>1:1 allocation ratio in blocks of 10	Stated<br>simple randomization, 3:3:2	Stated<br>simple randomization
Who generated the random allocation sequence, who enrolled participants, and who assigned participants to interventions	–	–	Partly stated<br>randomization: computer system	Stated<br>randomization: one author enrollment & assignment: study staff	Partly stated<br>randomization: computer system
If done, who was blinded after assignment to interventions	Partly stated<br>participants were blinded, not mentioned whether researchers were blinded	–	–	Stated<br>research team was partly blinded, participants were blinded	Partly stated<br>participants were not blinded, not mentioned whether researchers were blinded

For each group, the numbers of participants who were randomly assigned, received intended treatment, and were analyzed for the primary outcome	Stated	Stated	Stated	Stated	Stated
For each group, losses and exclusions after randomization, together with reasons	Stated	Partly stated	Partly stated<br>reasons for drop-outs unknown	Stated	Partly stated<br>reasons for drop-outs unknown
A table showing baseline demographic and clinical characteristics for each group	Yes	Yes	Yes	Yes	Yes
For each primary and secondary outcome, results for each group, and the estimated effect size and its precision (such as 95% confidence interval)	Partly stated<br>no CIs for effect sizes	Partly stated<br>no CIs for effect sizes	Stated	Partly stated<br>no CIs for effect sizes	Partly stated<br>no CIs for effect sizes
Results of any other analyses performed, including subgroup analyses and adjusted analyses, distinguishing pre-specified from exploratory	No other analyses performed	Stated	No other analyses performed	Stated	Stated
Trial limitations, addressing sources of potential bias, imprecision, and, if relevant, multiplicity of analyses	- sample size was not calculated → might be underpowered<br>- due to short intervention period and no follow-up assessments possible effects might not have been observed<br>- no preregistration	- while sample size calculations were mentioned, it remains unclear to which measure the effect size refers to<br>- not stated whether anyone was blinded<br>- calculated sample size was not met<br>- preregistration lacks methods of statistical analyses → unclear if exploratory analyses were planned in advance → multiplicity possible	- no active control condition<br>- no sample size calculated (pilot study)<br>- small sample size → probably underpowered<br>- no preregistration	- sample size calculations were based on the effect size for stress reductions → applicability for well-being unclear<br>- due to short intervention period and no follow-up assessments possible effects might not have been observed<br>- preregistration lacks methods of statistical analyses → unclear if exploratory analyses were planned in advance →	- no sample size calculations → makes interpretation of results (effect sizes) non-transparent<br>- extremely high drop-out rate limits representativeness<br>- preregistration lacks methods of statistical analyses → unclear if they were planned in advance → multiplicity possible

		- offered incentives may have affected motivation of participants → low intrinsic motivation may have had impact on execution		multiplicity possible<br>- offered incentives may have affected motivation of participants → low intrinsic motivation may have had impact on execution	
Generalizability (external validity, applicability) of the trial findings	- mostly well-educated females → generalizability further limited	- sample mainly consistent of highly educated females which might limit external validity	- validity of scales not stated (in exact values), which restricts evaluation of generalizability<br>- gender and race homogeneity limits generalizability further	- Ecological Momentary Assessment of State Positive and Negative Affect: no validity values → hinders comparability<br>- sample consisted of above average stressed adults → limits generalizability	- limited as younger participants more likely to drop-out<br>- highly educated sample → further limits generalizability
Interpretation consistent with results, balancing benefits and harms, and considering other relevant evidence	- it seems unreasonable to conclude that happiness seekers significantly benefit from mindfulness-based interventions when only one out of four aspects was found to be significant<br>- results impeded into prior literature	- interpretation consistent with results<br>- results embedded into prior literature	- even though the intervention group showed significant improvements in their positive mental health from pre- to post-intervention, contradictory results were found from pre- to mid-intervention → stated conclusion limited<br>- caution needs to be addressed when interpreting results as a 90% confidence interval used which increases risks of Type I error<br>- results impeded into prior literature	- interpretation consistent with results<br>- results embedded into prior literature	- interpretation consistent with results<br>- results embedded into prior literature
Registration number and name of trial registry	–	Stated<br>ClinicalTrials.gov (NCT04936893)	–	Stated<br>clinicaltrials.gov (02433431)	Stated<br>Chinese Clinical Trial Registry (13003468)
Where the full trial protocol can be accessed, if available	–	–	–	–	–

Selected CONSORT Statement criteria	Noone and Hogan [52], (2018)	Ponzo et al [53], (2020)	Robinson [54], (2018)	Schulte-Frankenfeld and Trautwein [55], (2021)	Smith et al [56], (2020)
Identification as a randomized trial in the title	Yes	Yes	No	Yes	Yes
Structured summary of trial design, methods, results, and conclusions	Yes	Yes	No	No	No
Specific objectives or hypotheses	Stated	Stated	Stated	Stated	Partly stated
Eligibility criteria for participants	Inclusion and exclusion criteria stated	Inclusion and exclusion criteria stated	Not stated	Inclusion and exclusion criteria stated	Not stated
The interventions for each group with sufficient details to allow replication, including how and when they were actually administered	Yes	Yes	Yes	Yes	Yes
Completely defined pre-specified primary and secondary outcome measures, including how and when they were assessed	Stated	Stated	Partly stated<br>no differentiation between primary and secondary outcomes	Stated	Stated
How sample size was determined	Stated	Stated	–	Not stated<br>indicative sensitivity analysis after convenience sampling	–
Method used to generate the random allocation sequence	–	Stated<br>R (Minirand package)	–	Stated<br>randomization software	–
Type of randomization, details of any restriction	Stated<br>block randomization with 1:1 ratio and blocks of 6	–	–	Stated<br>1:1 group allocation ratio	–
Who generated the random allocation sequence, who enrolled participants, and who assigned participants to interventions	Partly stated<br>assignment: researchers	–	–	–	–
If done, who was blinded after assignment to interventions	Stated<br>participants and researchers were blinded	Stated<br>neither participants nor researchers were blinded	–	Partly stated<br>blinded randomization	–

For each group, the numbers of participants who were randomly assigned, received intended treatment, and were analyzed for the primary outcome	Stated	Stated	Stated	Stated	Stated
For each group, losses and exclusions after randomization, together with reasons	Stated	Stated	Stated	Stated	Partly stated<br>Reasons not always mentioned
A table showing baseline demographic and clinical characteristics for each group	Barely given	Barely given	No	Yes	No
For each primary and secondary outcome, results for each group, and the estimated effect size and its precision (such as 95% confidence interval)	Partly stated<br>no effect sizes	Partly stated<br>no CIs for effect sizes	Partly stated<br>no CIs for effect sizes	Partly stated<br>no CIs for effect sizes	Stated
Results of any other analyses performed, including subgroup analyses and adjusted analyses, distinguishing pre-specified from exploratory	Partly stated	Stated	No other analyses performed	Stated	No other analyses performed
Trial limitations, addressing sources of potential bias, imprecision, and, if relevant, multiplicity of analyses	- sample size calculations were based on the effect size for mindfulness → applicability for well-being unclear<br>- due to attrition the calculated sample size was not reached at follow-up → might be underpowered<br>- small sample size<br>- offered incentives may have affected motivation of participants → low intrinsic motivation may have had	- no active control condition<br>- unclear to which effect the power analysis refers to → applicability for well-being unclear<br>- offered incentives may have affected motivation of participants → low intrinsic motivation may have had impact on execution	- very small sample size<br>- no active control condition<br>- no preregistration<br>- no a prior sample size calculation was performed	- no active control condition<br>- no a prior sample size calculation was performed<br>- small sample size<br>- no preregistration → unclear whether all analyses were planned in advance → multiplicity possible	- no active control condition<br>- no preregistration<br>- no a prior sample size calculation was performed

	impact on execution<br>- preregistration lacks methods of secondary statistical analyses → unclear if they were planned in advance → multiplicity possible				
Generalizability (external validity, applicability) of the trial findings	- external validity limited due to sample consisting solely of university students	- lack of data on ethnicity and other demographics hinders generalizability	- the small sample size makes applicability for broader population difficult<br>- generalizability limited due to predominantly female sample	- sample mainly consistent of highly educated females which might limit external validity	- not clear which aspect of the intervention contributes to beneficial outcomes as the intervention contains multiple components → might not be due to mindfulness training<br>- study was conducted across seven U.S. cities → good applicability
Interpretation consistent with results, balancing benefits and harms, and considering other relevant evidence	- interpretation consistent with results<br>- results embedded into prior literature	- interpretation consistent with results<br>- results partly embedded into prior literature	- interpretation not consistent with results (increased positive affect in treatment group over time was reported, even though F-test did not reach statistical significance)<br>- results embedded into prior literature	- presentation of results non-transparent → significant time effect of total life satisfaction was never mentioned in text but stated in table<br>- conclusion incorrect → stated improvement of mental well-being neither visible in results nor discussed in text<br>- perceived stress was a global indicator of mental well-being → unusual definition<br>- results embedded into prior literature	- interpretation consistent with results<br>- results embedded into prior literature
Registration number and name of trial registry	Stated<br>AEA Social Science Registry<br>(0000756)<br>and retrospectively:<br>ISRCTN registry<br>(16588423)	Stated<br>Open Science Framework (OSF.io) 2zd45;<br>https://osf.io/2zd45/	–	–	–

Where the full trial protocol can be accessed, if available	Stated<br>Noone C, Hogan MJ. A protocol for a randomised active-controlled trial to evaluate the effects of an online mindfulness intervention on executive control, critical thinking and key thinking dispositions in a university student sample. BMC Psychol. 2016;4:1–12. https://doi.org/10.1186/s40359-016-0122-7	–	–	–	–

Selected CONSORT Statement criteria	Taylor et al [57], (2022)	Thabrew et al [58], (2022)	Vu [59], 2018 (pilot study)	Vu [59], (2018)	Walsh et al [33], (2019)
Identification as a randomized trial in the title	Yes	Yes	Yes	Yes	Yes
Structured summary of trial design, methods, results, and conclusions	Yes	Yes	No	No	Yes
Specific objectives or hypotheses	Stated	Stated	Stated	Stated	Stated
Eligibility criteria for participants	Inclusion and exclusion criteria stated	Inclusion and exclusion criteria stated	Inclusion criteria only	Inclusion criteria only	Inclusion criteria only
The interventions for each group with sufficient details to allow replication, including how and when they were actually administered	Yes	Partly stated, number of sessions and duration per day unknown	Partly stated sessions and duration per day unknown	Partly stated sessions and duration per day unknown	Yes
Completely defined pre-specified primary and secondary outcome measures, including how and when they were assessed	Stated	Stated	Partly stated no differentiation between primary and secondary outcomes	Stated	Partly stated no differentiation between primary and secondary outcomes
How sample size was determined	Stated	Stated	Stated	Stated	Stated
Method used to generate the random allocation sequence	Stated Qualtrics	Stated web application	Stated random generator	Stated random generator	–
Type of randomization, details of any restriction	Stated block randomization (1:1, block size of 4)	–	–	–	–
Who generated the random allocation sequence, who enrolled participants, and who assigned participants to interventions	Partly stated randomization: Qualtrics	–	–	Partly stated randomization and assignment: lead author (stated in Appendix)	–
If done, who was blinded after assignment to interventions	Stated participants were blinded, parts of the research team were blinded	Stated neither participants nor researchers were blinded	–	Partly stated participants were blinded, not mentioned whether researchers were blinded	–

For each group, the numbers of participants who were randomly assigned, received intended treatment, and were analyzed for the primary outcome	Stated	Stated	Stated	Stated	Stated
For each group, losses and exclusions after randomization, together with reasons	Partly stated<br>reasons for drop-outs unknown	Stated	Partly stated<br>reasons for drop-outs after using the app unknown	Stated	Stated
A table showing baseline demographic and clinical characteristics for each group	Yes	Yes	No	Yes<br>in Appendix	No
For each primary and secondary outcome, results for each group, and the estimated effect size and its precision (such as 95% confidence interval)	Partly stated<br>no CIs for effect sizes	Partly stated<br>no CIs for effect sizes	Partly stated<br>no CIs for effect sizes	Partly stated<br>no CIs for effect sizes	–
Results of any other analyses performed, including subgroup analyses and adjusted analyses, distinguishing pre-specified from exploratory	Stated	Stated	No other analyses performed	Stated	Stated
Trial limitations, addressing sources of potential bias, imprecision, and, if relevant, multiplicity of analyses	- sample size calculations were based on effect sizes for stress → applicability for well-being unclear<br>- exploratory analyses and general linear mixed model were not preregistered → multiplicity possible<br>- differences in content delivery makes digital placebo effect possible	- no active control condition<br>- due to attrition the calculated sample size was not reached at follow-up → might be underpowered	- calculated sample size was not reached → study underpowered<br>- no specification regarding where effect size was retrieved from and which measure it belongs to<br>- no preregistration	- sample size calculations were based on effect sizes for stress and anxiety → applicability for well-being unclear<br>- blinding may not have worked as participants were classmates that communicated with each other<br>- no preregistration → unclear whether all analyses were planned in advance → multiplicity possible	- sample size calculations: no specification regarding where effect size was retrieved from and which measure it belongs to<br>- long term effect undetectable due to lack of follow-up assessments<br>- preregistration lacks methods of statistical analyses → unclear if exploratory analyses were planned in advance → multiplicity possible<br>- offered incentives may

					have affected motivation of participants → low intrinsic motivation may have had impact on execution
Generalizability (external validity, applicability) of the trial findings	- generalizability limited do to very specific sample (health care workers)	- generalizability limited to people aged 16 to 30<br>- generalizability limited due to predominantly female sample<br>- no instruction on frequency of app usage	- generalizability limited due to predominantly female sample<br>- very small sample size further limits generalizability<br>- no instruction on frequency of app usage	- external validity limited due to sample consisting solely of university students<br>- no instruction on frequency of app usage	- no validity details of used scales which restricts generalizability<br>- mostly female participants<br>- app only available for iOS devices → generalizability further limited
Interpretation consistent with results, balancing benefits and harms, and considering other relevant evidence	- well-being measures were presented in table but not interpreted in text<br>- results impeded into prior literature	- interpretation consistent with results<br>- results embedded into prior literature	- non-transparency of results: p-value .051 is presented as .05 in Table 1<br>- misleading presentation of results as p=.051 was phrased as trending towards significance<br>- non-significant result was interpreted as significant<br>- results not embedded into prior literature	- inconsistency in reported results: p-values not continuously stated which makes interpretation and comparison difficult<br>- results embedded into prior literature	- interpretation of trait well-being measures inconsistent with results (non-significance was interpreted as a trend towards desired direction)<br>- results embedded into prior literature
Registration number and name of trial registry	Stated<br>International Standard Randomised Controlled Trial Number ISRCTN15424185	Stated<br>Australian New Zealand Clinical Trials Registry (ACTRN12620000516987)	–	–	Stated, retrospectively clinicaltrials.gov (03783793)
Where the full trial protocol can be accessed, if available	–	Stated<br>Serlachius A, Schache K, Boggiss A, et al. Coping skills mobile APP to support the emotional well-being of young people during the COVID-19 pandemic: protocol for a mixed methods study.	–	–	–

Selected CONSORT Statement criteria	Xu et al [60], (2022)	Yang et al [61], (2018)	Yoon et al [62], (2022)
Identification as a randomized trial in the title	Yes	Yes	Yes
Structured summary of trial design, methods, results, and conclusions	Yes	Yes	Yes
Specific objectives or hypotheses	Stated	Stated	Stated
Eligibility criteria for participants	Inclusion and exclusion criteria stated	Exclusion criteria only	Inclusion and exclusion criteria stated
The interventions for each group with sufficient details to allow replication, including how and when they were actually administered	Partly stated<br>control group received no instruction	Yes	Yes
Completely defined pre-specified primary and secondary outcome measures, including how and when they were assessed	Stated	Partly stated<br>no differentiation between primary and secondary outcomes	Stated
How sample size was determined	Stated	–	Stated
Method used to generate the random allocation sequence	Stated<br>randomization website	Stated<br>computer-generated randomization program	Stated<br>random number table of 20×40 cells generated by a computer
Type of randomization, details of any restriction	Stated<br>1:1 ratio	–	Stated<br>1:1 allocation ratio
Who generated the random allocation sequence, who enrolled participants, and who assigned participants to interventions	–	–	Stated<br>one investigator enrolled participants, another generated random number table and assigned participants
If done, who was blinded after assignment to interventions	Stated<br>Participants were not blinded, data analysists were blinded	–	Stated<br>no one was blinded

For each group, the numbers of participants who were randomly assigned, received intended treatment, and were analyzed for the primary outcome	Stated	Partly stated<br>no information regarding group sizes after drop-outs	Stated<br><br>Partly stated<br>no reasons for drop-out
For each group, losses and exclusions after randomization, together with reasons	Stated	Partly stated<br>no information regarding group sizes after drop-outs plus no reasons	Stated
A table showing baseline demographic and clinical characteristics for each group	Yes	Yes	Yes
For each primary and secondary outcome, results for each group, and the estimated effect size and its precision (such as 95% confidence interval)	Partly stated<br>effect sizes only for overall groups, no CIs for effect sizes	Partly stated<br>no effect sizes	Partly stated<br>no CIs for effect sizes
Results of any other analyses performed, including subgroup analyses and adjusted analyses, distinguishing pre-specified from exploratory	No other analyses performed	No other analyses performed	No other analyses performed
Trial limitations, addressing sources of potential bias, imprecision, and, if relevant, multiplicity of analyses	- no active control condition<br>- sample size calculations were based on the effect size for stress → applicability for well-being unclear<br>- preregistration lacks methods of statistical analyses	- no active control group<br>- no sample size calculations → makes interpretation of results (effect sizes) non-transparent<br>- small sample size → might be underpowered<br>- attrition rate neither stated nor calculable due to non-transparency of drop-outs<br>- did not control for personality traits that might have moderated the	- no active control condition<br>- while sample size calculations were mentioned, it remains unclear to which measure the effect size refers to<br>- small sample size → probably underpowered<br>- no blinding

		outcomes<br>- no preregistration → unclear whether all analyses were planned in advance → multiplicity possible	
Generalizability (external validity, applicability) of the trial findings	- sample retrieved from two emergency departments → limits generalizability	- limited generalizability due to very specific sample (medical students)	- sample consisted of stressed middle-aged adults → limits generalizability
Interpretation consistent with results, balancing benefits and harms, and considering other relevant evidence	- interpretation consistent with results<br>- results not embedded into prior literature → hinders comparability	- interpretation not consistent with results: an improvement in well-being was reported even though results did not reach significance<br>- conclusion not consistent with found results, as it is not clear that intervention improved well-being<br>- results not embedded into prior literature → hinders comparability	- inconsistent assessment of results in relation to prior studies<br>- results consistent with other recent studies<br>- results impeded into prior literature
Registration number and name of trial registry	Stated<br>Australian<br>New Zealand Clinical Trials Registry<br>(ACTRN 12619001175167)	–	Stated<br>Clinical Research Information Service<br>(KCT0006892)
Where the full trial protocol can be accessed, if available	–	–	–

Note. MM = mindfulness meditation; – = not reported. See main text for reference list.

Figure S1

Detailed overview of risk of bias assessment.

Ref.	D1	D2	D3	D4	D5	Overall
Bostock et al. (2019)	!	-	!	!	!	-
Champion et al. (2018)	!	!	-	!	+	-
Carissoli et al. (2017)	!	-	+	!	!	-
Coelhoso et al. (2019)	-	!	!	!	!	-
Deady et al. (2022)	+	!	!	!	+	!
Economides et al. (2018)	!	!	!	!	-	-
Flett et al. (2018)	!	-	!	!	-	-
Fuller-Tyszkiewicz et al. (2020)	!	!	!	-	+	-
Gnanapragasam et al. (2023)	+	!	+	!	+	!
Hirshberg et al. (2021)	-	!	+	!	-	-
Howells et al. (2016)	+	!	-	+	-	-
Keng et al. (2022)	!	!	+	!	-	-
Levin et al. (2022)	!	-	-	+	!	-
Lindsay et al. (2018)	+	!	+	+	-	-
Mak et al. (2018)	+	!	!	!	-	-
Noone& Hogan (2018)	+	+	!	+	-	-
Ponzo et al. (2020)	!	!	!	!	!	!
Robinson (2018)	!	-	+	!	!	-
Schulte-Frankenfeld & Trautwein (2021)	!	-	-	!	-	-
Smith et al. (2020)	!	-	!	!	!	-
Taylor et al. (2022)	+	!	!	+	-	-
Thabrew et al. (2022)	!	!	-	!	-	-
Vu pilot study (2018)	!	-	!	+	-	-
Vu (2018)	+	!	-	!	-	-
Walsh et al. (2019)	+	-	!	!	-	-
Xu et al. (2022)	+	!	!	!	!	!
Yang et al. (2018)	!	!	-	!	!	-
Yoon et al. (2022)	-	!	+	!	+	-

+ Low risk
! Some concerns
- High risk

D1 Randomisation process
D2 Deviations from the intended interventions
D3 Missing outcome data
D4 Measurement of the outcome
D5 Selection of the reported result
